# Dual‐Band Fano Resonance of Low‐Frequency Sound Based on Artificial Mie Resonances

**DOI:** 10.1002/advs.201901307

**Published:** 2019-08-20

**Authors:** Ye‐Yang Sun, Jian‐Ping Xia, Hong‐Xiang Sun, Shou‐Qi Yuan, Yong Ge, Xiao‐Jun Liu

**Affiliations:** ^1^ Research Center of Fluid Machinery Engineering and Technology Faculty of Science Jiangsu University Zhenjiang 212013 China; ^2^ State Key Laboratory of Acoustics Institute of Acoustics Chinese Academy of Sciences Beijing 100190 China; ^3^ Key Laboratory of Modern Acoustics Department of Physics and Collaborative Innovation Center of Advanced Microstructures Nanjing University Nanjing 210093 China

**Keywords:** acoustic Fano resonances, dual‐band, low‐frequency sounds, Mie resonances

## Abstract

It is reported both experimentally and numerically that dual‐band acoustic Fano resonances (AFRs) of low‐frequency sound are realized by a compound unit array composed of two types of multiple‐cavity unit cells with different inner radii. Eigenmode analyses show that two types of monopolar Mie resonance (MMR) modes can be observed below 650 Hz, which arise from the coupling resonance of the overall structure and the Helmholtz resonance of each resonance cavity, respectively. Based on the MMRs with the out‐of‐phase characteristic induced by the mutual coupling of the two types of unit cells, the dual‐band AFRs, in which the quality factor of the AFR II can exceed 600 when the ratio of the inner radii is closed to 1.0, can be observed. More interestingly, the application of the dual‐band AFRs in sound encryption communication is further discussed. The proposed multiple‐cavity unit cell and its associated dual‐band AFRs provide diverse routes to design multiband sound devices with versatile applications, such as filtering, sensing, and communication.

## Graphical abstract

**Dual‐band acoustic Fano resonances (AFRs) of low‐frequency sound** is experimentally realized based on a multiple‐cavity unit cell with two types of monopolar Mie resonance (MMR) modes. The dual‐band AFRs arise from the MMRs with an out‐of‐phase characteristic created by the coupling of the unit cells with different inner radii. Additionally, the application of the dual‐band AFRs in sound encryption communication is discussed in detail.

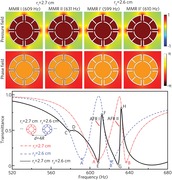

## 1 Introduction

A resonant peak with asymmetric profile and ultranarrow linewidth is known as Fano resonance, which is induced by the interference between a discrete resonance state and a smooth continuum state in background media. In the past decades, the Fano resonance has become a hot topic because of its important applications in the fields of switching,^1^, ^2^ biosensing,^3^, ^4^, ^5^ lasing,^6^ and slow‐light technology,^7^, ^8^ which need extremely high sensitivity or strong resonance and radiation states. The optical systems with the Fano resonance are usually constructed by complex unit cells with symmetry‐breaking structures, including asymmetric split ring resonators,^9^ dipole and quadrupole coupling metasurfaces and metamaterials,^3^, ^6^ and waveguide and plasmon coupling system.^4^

Inspired by the optical Fano resonances, the acoustic Fano resonance (AFR) has also given rise to increasing attentions because of its great potential in practical scenarios, such as filtering, sensing, and communication. In the past few years, several AFR systems based on the waveguides,^10^ concentric shells or pipes,^11^, ^12^ Helmholtz resonators,^13^, ^14^ and sonic crystals^15^, ^16^, ^17^, ^18^, ^19^ have been theoretically and experimentally realized. However, these structures are comparable to or larger than their wavelengths, especially the sonic crystals, which inevitably suffers from the large size for the low‐frequency sound. Additionally, the AFR effect only exists at a single working band in the previously demonstrated systems. The multiband AFR systems with subwavelength size and high quality factor (*Q*)‐factor for the low‐frequency sound still poses a significant challenge, which is very important for the development of multiband and multifunctional sound devices.

The emergence of acoustic metamaterials^20^, ^21^, ^22^, ^23^, ^24^, ^25^, ^26^, ^27^, ^28^, ^29^, ^30^ with subwavelength size and large negative refractive index has become a hot research topic due to its excellent functionality of acoustic manipulations. As a typical example, a maze‐like unit cell^31^ consisting of eight zigzag channels was designed to realize artificial Mie resonances.^32^ Based on the artificial Mie resonances, the high reflectance,^31^ rainbow trapping,^33^ and enhanced emission^34^ for the low‐frequency sound were theoretically and experimentally realized. Beyond that, by coupling the same maze‐like unit cells, the directivity^35^ and extraordinary transmission^36^ were obtained. However, few of them are concerned about the AFR with Mie resonances due to the difficulty of strong coupling between two types of unit cells with different structure parameters.

In this work, we propose a multiple‐cavity unit cell consisting of a central cavity surrounded by eight interconnected identical resonance cavities, which enables rich Mie resonance modes and has a diameter of 0.18λ and a filling ratio of 23%. It is observed that two types of monopolar Mie resonance (MMR) modes exist below 650 Hz, which are created by the coupling resonance of the overall structure and the Helmholtz resonance of each resonance cavity, respectively. Based on a composition unit cell composed of two multiple‐cavity unit cells with different inner radii, we can obtain the dual‐band AFRs with high quality factors which stems from the MMRs with the out‐of‐phase characteristic induced by the mutual coupling of the two unit cells. The measured results agree with the simulated ones. Finally, we discuss the feasibility of applying the dual‐band AFRs in sound encryption communication in detail.

## 2 Multiple‐Cavity Unit Cell of Mie Resonance

**Figure 1:**
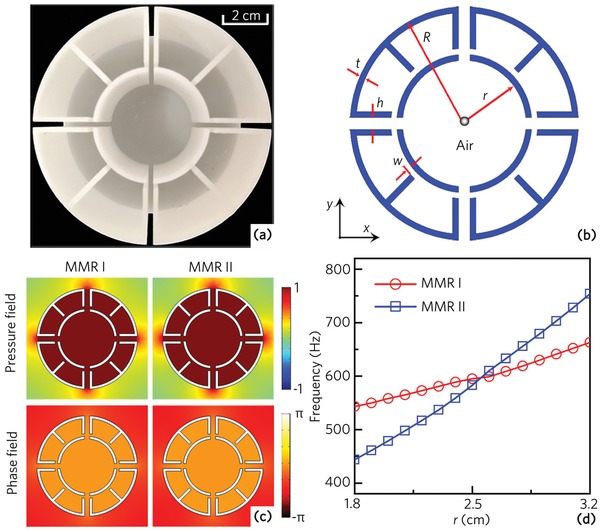
a) Photograph and b) schematic of a multiple‐cavity unit cell in *xy‐*plane. c) Simulated pressure and phase eigenfunctions of the multiple‐cavity unit cell with *r* = 2.7 cm at 609 and 633 Hz. The two eigenmodes are denoted as MMRs I and II. d) Eigenfrequencies of the MMRs I and II with different parameter *r*.

We propose a 2D multiple‐cavity unit cell which has Mie resonance characteristics. **Figure** **1**a shows the photograph of the multiple‐cavity unit cell fabricated with epoxy resin by mean of 3D printing, which consists of a central cavity surrounded by eight interconnected identical resonance cavities. The schematic cross‐sectional illustration of the unit cell is shown in Figure 1b, in which the thickness of solid frames is *t*, the width of four channels is *h*, the open width of eight cavities is *w*, and the inner and outer radii of the unit cell are *r* and *R*, respectively. Here, we adopt the finite element method based on COMSOL Multiphysics software to numerically simulate Mie resonance characteristics, and the structure parameters are selected as *t* = 2.6 mm, *h* = 5.0 mm, *w* = 2.5 mm, *R* = 5.0 cm and the inner radius *r* is tunable. Due to the existence of several slits in the multiple‐cavity unit cell, we consider the visco‐thermal loss by using Thermoviscous Acoustic‐Solid Interaction module throughout this work. The material parameters are used as follows: the density ρ = 1050 kg m^−3^, the Young's modulus *E* = 5.08 GPa, and the Poisson ratio *v* = 0.35 for epoxy resin; the density ρ = *p*_0_ *M*/*RT* and the acoustic velocity $c\;=\;\sqrt{\gamma RT\text{/}M}$ for air, in which γ = 1.4 is the ratio of the molar heat capacities of air, *M* = 28.97 × 10^−3^ kg mol^−1^ is the molar mass of air, *R* = 8.31 J (mol K^−1^)^−1^ is the molar gas constant, *p*_0_ = 101.325 kPa is the pressure at 273 K, and *T* = 293 K is the temperature of air.

Figure 1c shows the pressure amplitude and phase eigenfunctions of the multiple‐cavity unit cell with *r* = 2.7 cm at 609 and 633 Hz. It is found that the pressure and phase amplitudes are the same in the unit cell for both eigenmodes, showing typical features of MMR.^31^ Thus, in the proposed multiple‐cavity unit cell, we can simultaneously observe two types of MMR modes around 600 Hz, labeling as MMRs I and II. In addition to the MMR, other types of the Mie resonance modes, such as the dipole and quadrupole, can also be observed in the multiple‐cavity unit, which is shown in the Supporting Information. Figure 1d shows the eigenfrequencies of the MMR modes with different inner radius *r*. It is found that, with the increase of *r*, both MMR modes also exist, and their eigenfrequencies increase gradually. In addition to the inner radius *r*, the eigenfrequencies of the MMRs I and II are also closely related to the parameters *h*, *w*, and *R*, which is discussed in the Supporting Information.

**Figure 2:**
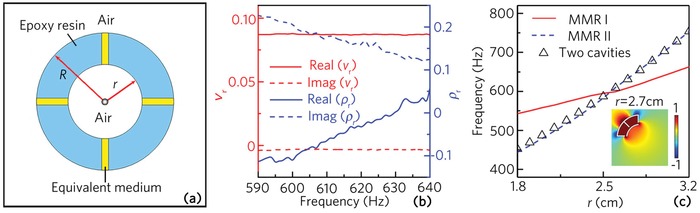
a) Schematic of the equivalent physical model of the multiple‐cavity unit cell, in which the inner and outer radii *r* and *R* are the same as those in Figure 1b. b) Relative equivalent velocity (*v*_r_) and density (ρ_r_) of the equivalent medium (four yellow channels in (a)). c) Eigenfrequencies of the MMRs I and II of the multiple‐cavity unit cells composed of with two and eight cavities with different values of *r*.

To analyze the mechanism of the MMRs I and II, we establish an equivalent physical model of the multiple‐cavity unit cell,^31^ which consists of four blue fan‐shaped regions and yellow straight channels filled with the epoxy resin and the equivalent medium, respectively (shown in **Figure** **2**a). Figure 2b shows the calculated relative equivalent velocity (*v*_r_) and density (ρ_r_) of the equivalent medium, which are normalized by those of air. It is found that at the eigenfrequencies around the MMRs I (609 Hz) and II (633 Hz), the real parts of *v*_r_ are only about 0.087, and their imaginary parts are almost negligible, which indicates that the equivalent velocity is much slower than that of air. Therefore, similar to that of the maze‐like unit cell with eight zigzag channels,^31^ the MMRs I and II are also closely related to the ultraslow velocity of the equivalent medium.

To provide a further insight into the physical mechanism of both MMRs, we simulate the eigenfrequencies of two types of unit cells composed of the two and eight resonance cavities with different values of *r* (shown in Figure 2c), in which the other structure parameters of both unit cells are the same as those in Figure 1b. It is found that, for the unit cell composed of the two cavities, it only has a single eigenfrequency with different *r* which is very close to that of the MMR II. Additionally, the pressure distribution of its eigenmode shows typical characteristic of Helmholtz resonance (shown in the inset of Figure 2c). Beyond that, the pressure eigenfunctions and the eigenfrequencies of the unit cells composed of the four and six resonance cavities are also the same as those of the two cavities structure, which is displayed in the Supporting Information. Based on the aforementioned results, we demonstrate that the MMR II arises from the Helmholtz resonance of each resonance cavity, but the MMR I is attributed to the coupling resonance of the overall structure of the unit cell, which is similar to those of the maze‐like^31^ and ring‐shaped^37^ unit cells.

**Figure 3:**
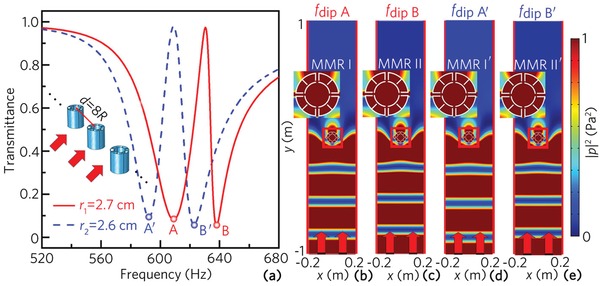
a) Transmittance spectra of two unit arrays composed of a single type of unit cell with *r*_1_ = 2.7 cm or *r*_2_ = 2.6 cm. The frequencies of the dips A, B, A′, and B′ are 608, 637, 592, and 622 Hz, respectively. Distributions of intensity field induced by the unit arrays with *r*_1_ = 2.7 cm at b) *f*_dipA_ and c) *f*_dipB_, and with *r*_2_ = 2.6 cm at d) *f*_dipA′_ and e) *f*_dipB′_. Red arrows and lines represent incident plane acoustic waves and periodic boundaries, respectively, and the upper side of the model is set as perfectly matched boundary to avoid reflected sound energy. Four insets at the upper side represent the zooms of the sound intensity distribution in four red squares.

**Figure** **3**a shows the transmittance spectra of two unit arrays with different values of *r*, in which the lattice constant is selected as *d* = 8*R*, and the other parameters are the same as those in Figure 1b. As shown in Figure 3a, there exist two sharp dips located at the points A and B for *r*_1_ = 2.7 cm (red solid line). With the decrease of the parameter *r*, both dips move to the low‐frequency region, and are located at the points A′ and B′ for *r*_2_ = 2.6 cm (blue dashed line). To show the formation mechanism of the dips, we simulate the corresponding intensity distributions at the four dips, which are shown in Figure 3b–e. Note that the incident waves cannot transmit through the unit arrays, and the acoustic intensity in the upper region is close to zero for the four cases. The upper insets are the zooms of the corresponding intensity distribution in the four red squares. Note that the MMRs I, II, I′, and II′ are excited, which corresponds to the dips A, B, A′, and B′, respectively. In addition, the frequencies of the four dips A, B, A′, and B′ are close to those of the MMRs of the unit cells with *r*_1_ = 2.7 cm and *r*_2_ = 2.6 cm (shown in Figure 1d), further indicating that these dips are created by the two MMR modes of the multiple‐cavity unit cell. Moreover, we also compare the transmittance spectra for *r*_1_ = 2.7 cm with and without visco‐thermal loss, which is shown in the Supporting Information. The result shows that the visco‐thermal loss has a minor effect on the two dips of the transmittance spectra.

## 3 Performance and Mechanism of Dual‐Band AFRs

**Figure 4:**
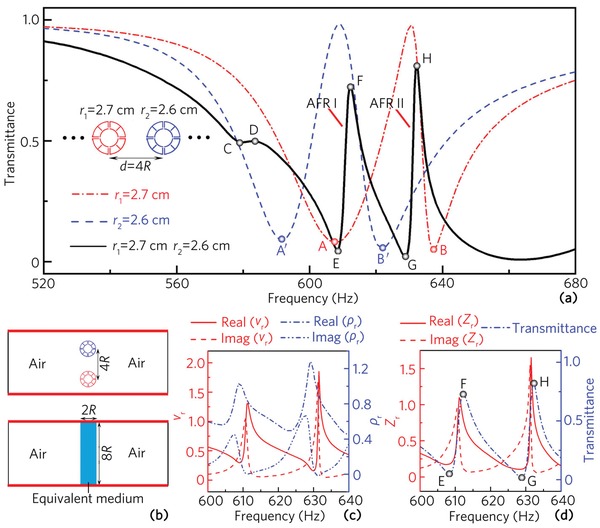
a) Transmittance spectrum of a compound unit array (black solid line) composed of two types of unit cells with *r*_1_ = 2.7 cm and *r*_2_ = 2.6 cm, and transmittance spectra of a unit array composed of a single type of unit cell with *r*_1_ = 2.7 cm (red dashed dot line) or *r*_2_ = 2.6 cm (blue dashed line) are also displayed for comparison. The frequencies of the dip E, peak F, dip G, and peak H are 608, 612, 629, and 632 Hz, respectively. b) Schematic of the compound unit array and its equivalent physical model. Red lines represent periodic boundaries. c) Relative equivalent velocity, density, d) impedance and transmittance spectrum of the equivalent medium.

Next, we simulate the transmittance spectrum of a compound unit array with the lattice constant *d* = 4*R* which consists of two types of unit cells with *r*_1_ = 2.7 cm and *r*_2_ = 2.6 cm (shown as the black line in **Figure** **4**a). Compared with the transmittance spectra of the unit array composed of a single type of unit cell with *r*_1_ = 2.7 cm or *r*_2_ = 2.6 cm, two unusual asymmetric peaks F and H can be observed for the compound unit array, showing typical dual‐band AFRs characteristics (denoting as AFRs I and II). Note that the asymmetric peaks F and H are located between the dips A and B′ and the dips B′ and B, respectively, and thus the AFRs I and II may arise from the mutual coupling of the MMRs I and II′ and the MMRs II and II′ induced by the unit cells with *r*_1_ and *r*_2_. Moreover, there also exist a dip C and a peak D around 580 Hz, but the AFR characteristic is not obvious because of the visco‐thermal loss of the unit cells, and the comparisons between the transmittance spectra with and without the visco‐thermal loss are shown in the Supporting Information. The results show that the visco‐thermal loss has a minor effect on the peaks F and H, but has a strong influence on the dip C and peak D. This is because the dip C and peak D arise from the mutual coupling of the MMRs I and I′ (corresponding to the dips A and A′ in Figure 3a), but have nothing to do with the MMRs II and II′. To quantify the performances of the dual‐band AFRs, we calculate the quality factor *Q* = *f*_0_/(*f*_peak_ − *f*_dip_) of the AFRs I and II,^38^ in which *f*_dip_ and *f*_peak_ are the frequencies of the dip E (dip G) and peak F (peak H), respectively, and *f*_0_ is the center frequency. The values of *Q* are calculated as 153 and 210 for the AFRs I and II, showing high performances of the dual‐band AFRs.

To explain the two sharp peaks F and H, we also introduce an effective medium of the compound unit array (shown as the blue region in Figure 4b), in which the effective parameters are theoretically calculated based on the complex reflection and transmission coefficients of the compound unit cell.^39^ The relative equivalent velocity (*v*_r_), density (ρ_r_), and impedance (*Z*_r_) of the equivalent medium are presented in Figure 4c,d. As shown in Figure 4d, the relative equivalent impedances of the peaks F and H are 0.86 + 0.14*i* and 0.97 + 0.10*i*, respectively. The real and imaginary parts of both equivalent impedances are close to 1.0 and 0, respectively, which demonstrates the existence of the peaks F and H. However, the transmittances of the peaks F and H cannot get close to 1.0. This is because their imaginary parts are not negligible due to the visco‐thermal loss of the unit cell.

**Figure 5:**
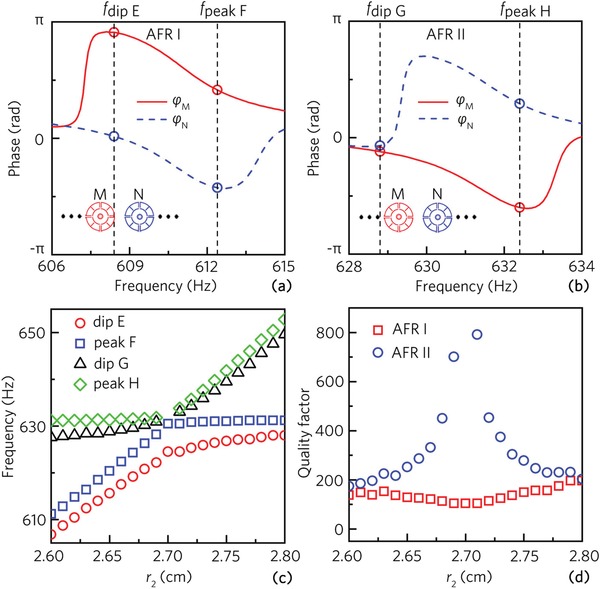
Phase spectra at the center of two types of unit cells (points M and N) with *r*_1_ = 2.7 cm and *r*_2_ = 2.6 cm in the compound unit array in the frequency ranges around the AFRs a) I and b) II. c) Frequencies of the dip E, peak F, dip G, and peak H, and d) quality factors of the AFRs I and II for the compound unit array with different values of *r*_2_.

To show the physical mechanism of the dual‐band AFRs, we simulate the phase spectra at the center of the two types of unit cells (points M and N), which is shown in **Figure** **5**a,b. It is found that, in the frequency ranges of the AFRs I (606–615 Hz) and II (628–634 Hz), most phase differences between the points M (ϕ_M_) and N (ϕ_N_) almost remains a constant of π. It is, therefore, demonstrated that the dual‐band AFRs arise from the mutual coupling of the MMR in the two types of unit cells with the out‐of‐phase characteristic.

Furthermore, we simulate the frequencies of the dip E, peak F, dip G, and peak H with different values of *r*_2_, and the parameter *r*_1_ = 2.7 cm remains constant. As shown in Figure 5c, when the value of *r*_2_ is close to that of *r*_1_, the frequency difference between the dip G and peak H decreases gradually, but that between the dip E and peak F increases slightly. In addition, when *r*_2_/*r*_1_ = 1.0, the dip G and peak H disappear simultaneously, and only the dip E and peak F exist. Therefore, the AFR II does not exist with *r*_2_/*r*_1_ = 1.0. To clearly show this phenomenon, we simulate the transmittance spectra for different values of *r*_2_/*r*_1_ around 1.0, which is displayed in the Supporting Information. Figure 5d shows the quality factor *Q* of the AFRs I and II with different values of *r*_2_. When the *r*_2_ is close to *r*_1_, the value of *Q* for the AFR II increases gradually and is larger than 600 when *r*_2_ = 2.69 and 2.71, and the AFR II does not exist with *r*_2_ = 2.70. However, the value of *Q* decreases slightly for the AFR I. Therefore, we can obtain the AFR with the high quality factor by selecting unit cells with similar inner radii.

## 4 Experimental Demonstration

**Figure 6:**
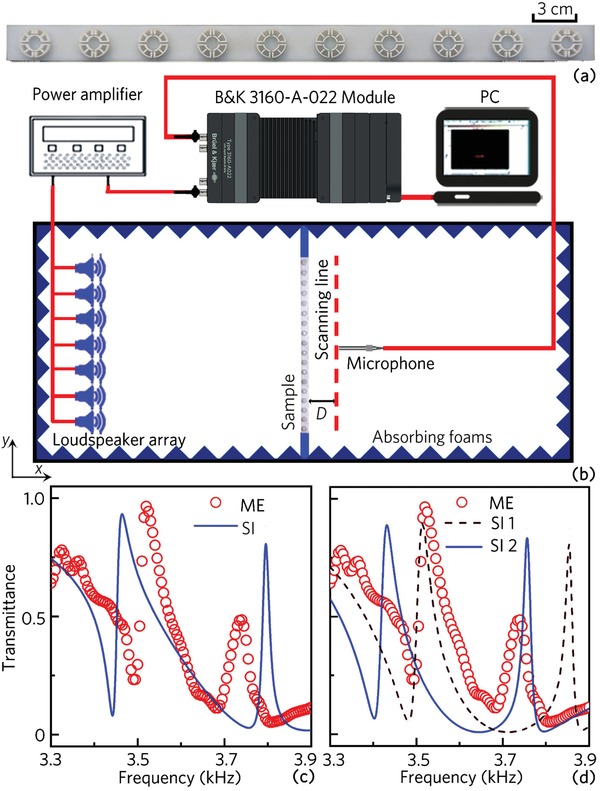
a) Photograph of half part of the sample and b) experimental set‐up. c) Measured (ME) and simulated (SI) transmittance spectra of the sample. d) Comparison between the experiment measurements and the simulated results for two different parameters of the unit cells (SI 1: *r*_m_ = 0.99*r*, *h*_m_ = 0.93*h* and *R*_m_ = 0.99*R*; SI 2: *r*_m_ = 0.99*r*, *h*_m_ = 0.93*h* and *R*_m_ = 1.00*R*).

To experimentally verify the dual‐band AFRs, we measure the transmittance spectra of a compound unit array, and the photograph of the compound unit array and the experiment set‐up are shown in **Figure** **6**a,b, respectively. In the experimental measurement, the compound unit array needs a certain number of periods to obtain better results, and thus the size of the experiment platform cannot satisfy this requirement. In addition, the overall scaling of the unit structure may cause considerable fabrication complexities because of thin frames of the unit cell. Based on these two factors, the parameters of the sample are selected as *t* = 0.6 mm, *h* = 1.5 mm, *w* = 0.6 mm, *R* = 10.0 mm, *r*_1_ = 5.0 mm, and *r*_2_ = 4.5 mm, and the unit period of sample is selected as 10. Figure 6c shows the measured (red open circles) and simulated (blue solid line) transmittance spectra, in which both results show the characteristics of the dual‐band AFRs. The measured quality factors of the AFRs I and II are 281 and 136, respectively, which are close to the simulated results (313 and 188 for the AFRs I and II). However, the measured frequency ranges of the AFRs I and II are not consistent with the simulated ones. This is because the performance of the dual‐band AFRs is sensitive to the geometrical parameters of the unit cells, and the structure errors of each unit cell in the sample are different because of the fabrication accuracy of 3D printing (about ±0.1 mm) and the fabrication complexities of the unit cells with circular shapes.

To further demonstrate it, we carefully measure the structure parameters of each unit cell in the sample, and the measured three important parameters of the unit cell *r*_m_, *h*_m_, and *R*_m_ are about in the ranges 0.97–1.00 *r*, 0.89–1.01 *h*, and 0.98–1.01 *R*, respectively. By selecting the parameters *r*_m_ = 0.99 *r*, *h*_m_ = 0.93 *h*, and *R*_m_ = 0.99 *R*, the corresponding simulated transmittance spectrum are shown as the brown dashed line in Figure 6d. Note that the frequency range of the AFR I is close to that of the measured result. In addition, by selecting the parameters *r*_m_ = 0.99 *r*, *h*_m_ = 0.93 *h*, and *R*_m_ = 1.00*R* (shown as the blue solid line), the frequency range of the AFR II is close to that of the measured result. Therefore, we demonstrate that the frequency bands of the AFRs I and II are very sensitive to the parameters of the unit cells, and the error of the measured frequency bands of the AFRs I and II arise from the structure errors of each multiple‐cavity unit cell in the sample.

## 5 Application

**Figure 7:**
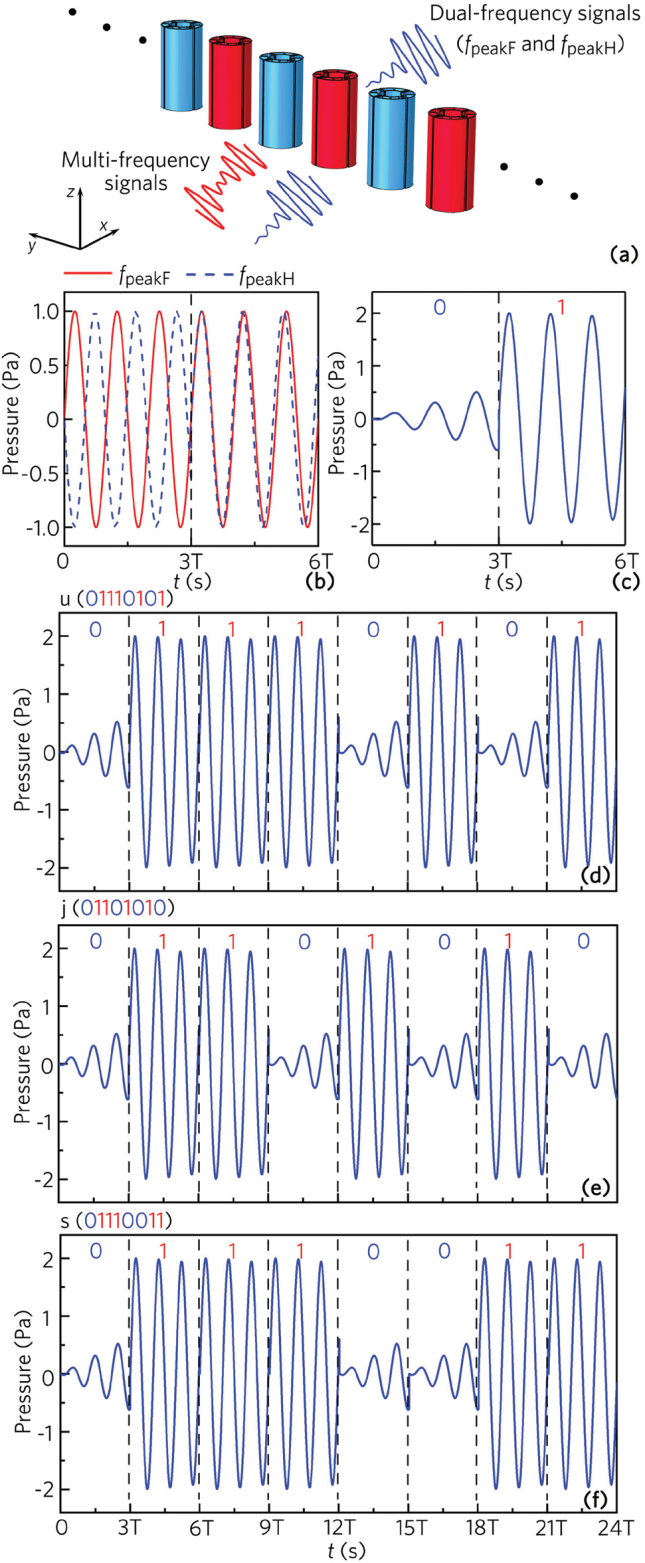
a) Schematic of the application of dual‐band AFRs in sound encryption communication. b) Input time‐domain signals at *f*_peakF_ and *f*_peakH_ for the out‐of‐phase (0–3 T) and in‐phase (3–6 T) cases. c) Linear interferences of output time‐domain signals at *f*_peakF_ and *f*_peakH_ for the out‐of‐phase (0–3 T) and in‐phase (3–6 T) cases. Three periods of output signals for the out‐of‐phase and in‐phase cases represent the codes “0” and “1”. Output time‐domain signals for the letters d) u (0 111 0101), e) j (0 110 1010), and f) s (0 111 0011).

The proposed dual‐band AFRs has potential applications in the sound encryption communication based on their two peaks F and H. As shown in **Figure** **7**a, the input multifrequency time‐domain signals which include two signals at *f*_peakF_ and *f*_peakH_ impinge on the compound unit array, and only the signals at *f*_peakF_ and *f*_peakH_ can pass through the unit array owing the two sharp peaks in the dual‐band AFRs. To realize the modulation of the output signals, we introduce two signals at *f*_peakF_ and *f*_peakH_ with the out‐of‐phase or in‐phase characteristics into the input multifrequency signals, which is shown in the ranges 0–3 and 3–6 T of Figure 7b (T is a signal period), respectively. By using three periods of two signals with the out‐of‐phase characteristic, we can obtain the output signal “0” based on the interference cancellation (shown in the range 0–3 T of Figure 7c). However, for the two signals with the in‐phase characteristic, the other output signal “1” is obtained due to the interference enhancement (shown in the range 3–6 T of Figure 7c). Therefore, by modulating the phases of the two input signals, we can realize the sound encryption communication. As an example, we use the word “ujs” to display this application. In the American standard code for information interchange, the binary forms of the letters “u”, “j,” and “s” are represented as 0 111 0101, 0 110 1010, and 0 111 0011. We can realize the sound communication of a single letter by using 24 signal periods. The output time‐domain signals for the letters “u”, “j,” and “s” are shown in Figure 7d–f, respectively. Thus, the potential application about the sound encryption communication based on the dual‐band AFRs is feasible.

## 6 Conclusion

In conclusion, we have realized dual‐band AFRs based on a multiple‐cavity unit cell which is composed of a central cavity surrounded by eight interconnected identical resonance cavities. The results show that the MMRs I and II exist below 650 Hz, which arise from the coupling resonance of the overall structure and the Helmholtz resonance of each resonance cavity of the unit cell, respectively. The eigenfrequencies of the MMRs I and II are mainly determined by the parameters *r*, *h*, and *R*. By combining the two types of unit cells with *r*_1_ = 2.7 cm and *r*_2_ = 2.6 cm, we can observe the dual‐band AFRs with high quality factors which arise from the MMRs with the out‐of‐phase characteristic induced from the mutual coupling of the two unit cells. Besides, the higher quality factor can be realized by selecting the similar values of *r*_2_ and *r*_1_, and is larger than 600 for the AFR II when the parameter *r*_2_/*r*_1_ is closed to 1.0. Moreover, we also experimentally demonstrate the dual‐band AFRs, in which the measured quality factors are very close to the simulated ones. The proposed multiple‐cavity unit cell has the advantages of a 0.18λ‐diameter, 23%‐filling ratio structure, and rich Mie resonance modes, which provide a fertile ground for designing novel multiband and multifunctional sound devices with versatile applications. Prospective applications of the dual‐band AFRs in sound encryption communication can be anticipated in the near future.

## 7 Experimental Section

The sample was placed at the middle of the experimental platform (shown in Figure 6b). A loudspeaker array was located at the left side of the sample to generate incident acoustic waves. A 0.25‐in‐diameter Brüel&Kjær type‐4961 microphone was adopted to detect acoustic pressure amplitudes, and the distance *D* between the sample and the scanning line was 5 cm. The sound absorbing foams were used to avoid reflected acoustic energy. By using the software PULSE Labshop, the transmission was calculated by recording the pressure amplitudes with and without the sample, respectively.

## Conflict of Interest

The authors declare no conflict of interest.

## Supporting information

Supplementary
