# Supplementary material for: Dual‐Band Fano Resonance of Low‐Frequency Sound Based on Artificial Mie Resonances
Source: Adv Sci (Weinh). 2019 Aug 20;6(20):1901307. doi: 10.1002/advs.201901307 (PMC6794620; doi:10.1002/advs.201901307)
Supplement: Supplementary file 1 — Supplementary [file ADVS-6-1901307-s001.pdf]

Copyright WILEY-VCH Verlag GmbH & Co. KGaA, 69469 Weinheim, Germany, 2019.

ADVANCED SCIENCE Open Access

Supporting Information

for *Adv. Sci.,* DOI: 10.1002/advs.201901307

Dual-Band Fano Resonance of Low-Frequency Sound Based on Artificial Mie Resonances

Ye-Yang Sun, Jian-Ping Xia, Hong-Xiang Sun, Shou-Qi Yuan, Yong Ge, and Xiao-Jun Liu**

Supporting Information

Dual-band Fano resonance of low-frequency sound based on artificial Mie resonances

Ye-yang Sun, Jian-ping Xia, Hong-xiang Sun, Shou-qi Yuan, Yong Ge, and Xiao-jun Liu**

The file includes:

Other Mie resonance modes of the multiple-cavity unit cell

Eigenfrequencies of the MMRs I and II with different *h, w* and *R*

Pressure eigenfunctions and eigenfrequencies of the unit cells composed of the four and six resonance cavities

Transmittance spectra of the unit array composed of a single type of unit cell with and without the visco-thermal loss

Transmittance spectra of the compound unit array composed of two types of unit cells with and without the visco-thermal loss

Transmittance spectra of the compound unit arrays with different r_2/r_1

Figure S1. Simulated pressure and phase eigenfunctions of two types of dipolar and quadripolar Mie resonance modes for the multiple-cavity unit cell, a), e) dipole I(634Hz), b), f) quadrupole I(635Hz), c), g) dipole II (1403Hz), and d), h) quadrupole II (1587Hz).

Figure S2. Eigenfrequencies of the MMRs I and II with different a) *h*, b) *w* and c) *R*.

Figure S3. Simulated pressure eigenfunctions of the unit cells composed of the a), b) four (I and II) and c) six resonance cavities, corresponding to the eigenfrequencies of 631, 631 and 630 Hz, respectively. d) Eigenfrequencies of the unit cells composed of the two, four, six and eight resonance cavities with different values of *r*.

Figure S4. Transmittance spectra of the unit array composed of the unit cell (r=2.7 cm) with and without the visco-thermal loss.

Figure S5. Transmittance spectra of the compound unit array composed of two types of unit cells (r_1=2.7 cm and r_2=2.6 cm) with and without the visco-thermal loss.

Figure S6. Transmittance spectra of the compound unit arrays composed of two types of unit cells with a) r_2/r_1=0.99, b) r_2/r_1=1.00 and c) r_2/r_1=1.01.

Other Mie resonance modes of the multiple-cavity unit cell

Figure S1 shows the simulated pressure and phase eigenfunctions of the multiple-cavity unit cell. As shown in Figure S1a-d, the corresponding two types of dipolar and quadripolar Mie resonance modes can be observed in the multiple-cavity unit cell. Moreover, the corresponding phase eigenfunctions show typical characteristics of these Mie resonant modes (Figure S1e-h). The result further demonstrates that the multiple-cavity unit cell has rich Mie resonant modes.

Eigenfrequencies of the MMRs I and II with different *h, w* and *R*

Figure S2 shows the eigenfrequencies of the MMRs I and II with different parameters *h*, *w* and *R*. As shown in Figure S2a, b, with the increase of *h* and *w*, the eigenfrequencies of the MMRs I and II increase gradually. However, the eigenfrequencies of the MMRs I and II decrease with the increase of *R* (shown in Figure S2c).

Pressure eigenfunctions and eigenfrequencies of the unit cells composed of the four and six resonance cavities

Figure S3a-c shows the simulated pressure eigenfunctions of three types of unit cells composed of four and six resonance cavities, in which the other parameters are the same as those in Figure 1b. Note that, there also only exists a single eigenmode around 630Hz for the three cases. Figure S3d shows the eigenfrequencies for five types of unit cells (including two- and eight-cavities structures) with different values

of *r*. Similar to the result of the two-cavities structure (Figure 2c), the unit cells composed of the four and six cavities have a single eigenfrequency with different *r*, which is very close to that of the MMR II of the unit cell with eight cavities.

Transmittance spectra of the unit array composed of a single type of unit cell with and without the visco-thermal loss

To show the influences of the visco-thermal loss on the transmission characteristics, we simulate the transmittance spectra of the unit array with and without the visco-thermal loss, which is shown in the **Figure S4**. Compared with the result without visco-thermal loss (red dashed line), the spectrum waveform almost remains unchanged, but moves to the low frequency region with the visco-thermal loss (blue solid line). In addition, the transmittances of the peak and two dips change slightly. Therefore, we can deduce that the visco-thermal loss has a minor effect on the two dips created by both the MMR modes of the multiple-cavity unit cell.

Transmittance spectra of the compound unit array composed of two types of unit cells with and without the visco-thermal loss

Figure S5 shows the transmittance spectra of the compound unit array with and without the visco-thermal loss. Note that there exist three-band AFRs in the transmittance spectrum without visco-thermal loss (red dashed line). However, by introducing the visco-thermal loss (blue solid line), the AFR around the dip C and peak D does not exist. This is because the dip C and peak D stem from the mutual coupling of the MMRs I and I’ (corresponding to the dips A and A’ in Figure 3a), but

have nothing to do with the MMRs II and II'. Therefore, the visco-thermal loss has a great influence on the dip C and peak D created by the type of the MMR I. Moreover, the visco-thermal loss also has a minor effect on the peaks F and H, but it is weaker than that of the dip C and peak D. This is because the existence of the peaks F and H is closely related to the type of the MMR II. Therefore, the AFRs I and II also exist with the visco-thermal loss.

Transmittance spectra of the compound unit arrays with different r_2/r_1

Figure S6 shows the transmittance spectra of the compound unit arrays with different values of r_2/r_1. Compared with the result in Figure 4a, the peak H are closer to the peak F when the parameter r_2/r_1=0.99 and 1.01 (Figure S6a, c). Meanwhile, the quality factor Q of the AFR II becomes higher, but that of AFR I is almost unchanged. Furthermore, when the parameter r_2/r_1=1.00 (Figure S6b), there only exists the AFR I, and the dip G and peak H disappear simultaneously.

Figure S1

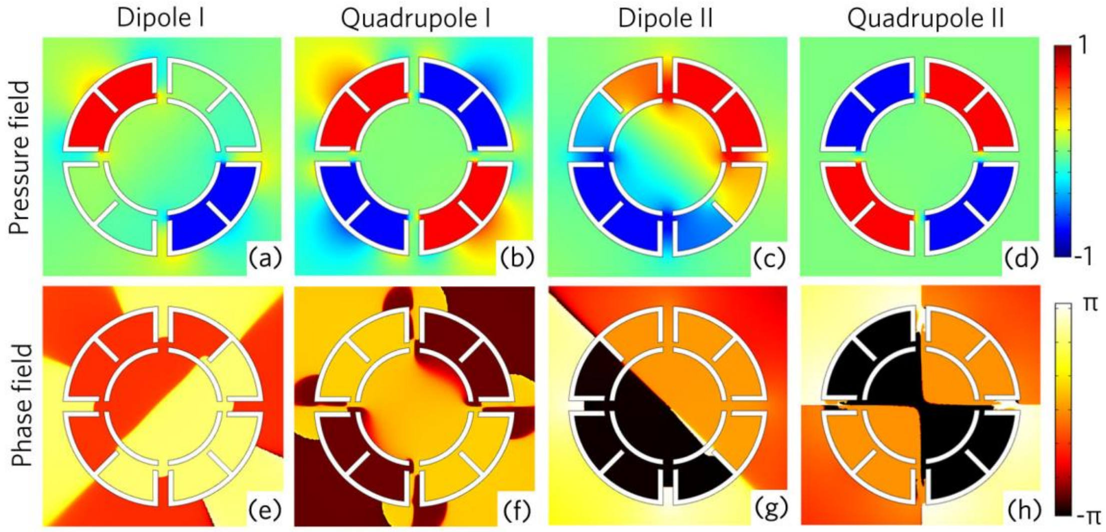

Figure S1. Simulated pressure and phase eigenfunctions of two types of dipolar and quadripolar Mie resonance modes for the multiple-cavity unit cell, a), e) dipole I (634Hz), b), f) quadrupole I (635Hz), c), g) dipole II (1403Hz), and d), h) quadrupole II (1587Hz).

Figure S2

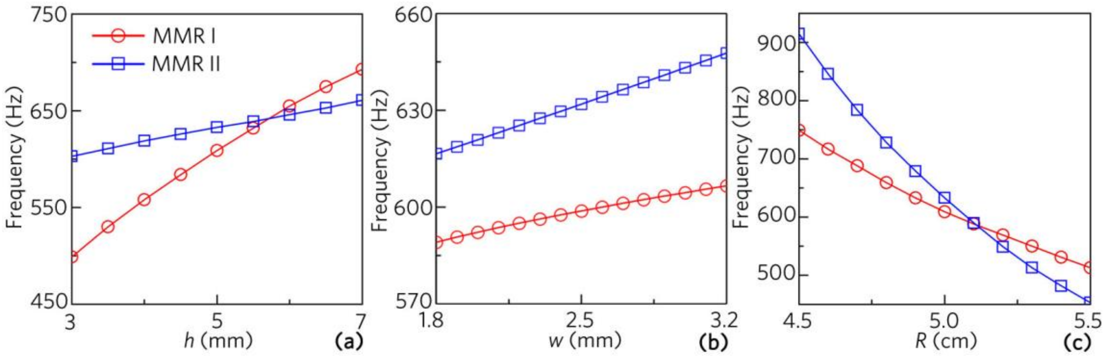

Figure S2. Eigenfrequencies of the MMRs I and II with different values of a) *h*, b) *w* and c) *R*.

Figure S3

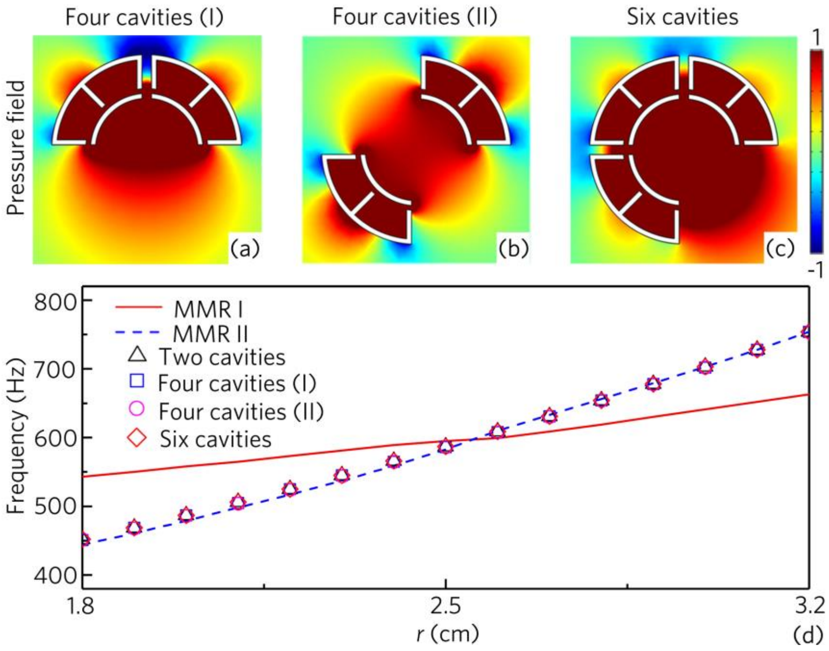

Figure S3. Simulated pressure eigenfunctions of the unit cells composed of the a), b) four (I and II) and c) six resonance cavities, corresponding to the eigenfrequencies of 631, 631 and 630 Hz, respectively. d) Eigenfrequencies of the unit cells composed of the two, four, six and eight resonance cavities with different values of *r*.

Figure S4

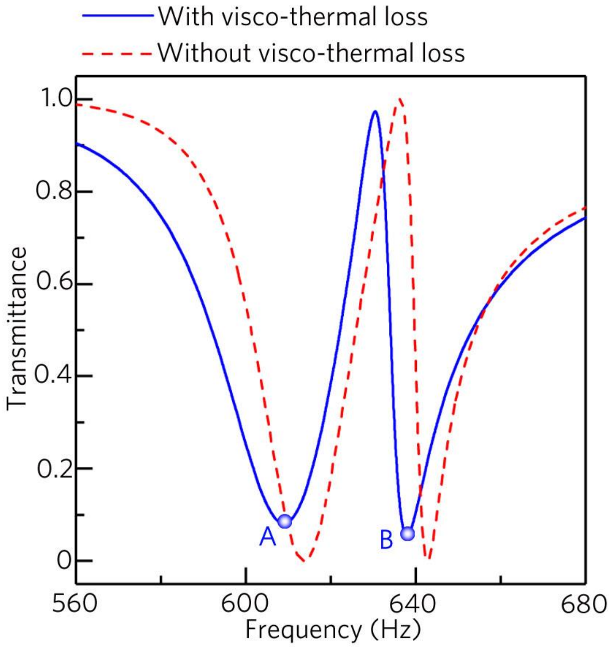

Figure S4. Transmittance spectra of the unit array composed of the unit cell (r=2.7 cm) with and without the visco-thermal loss.

Figure S5

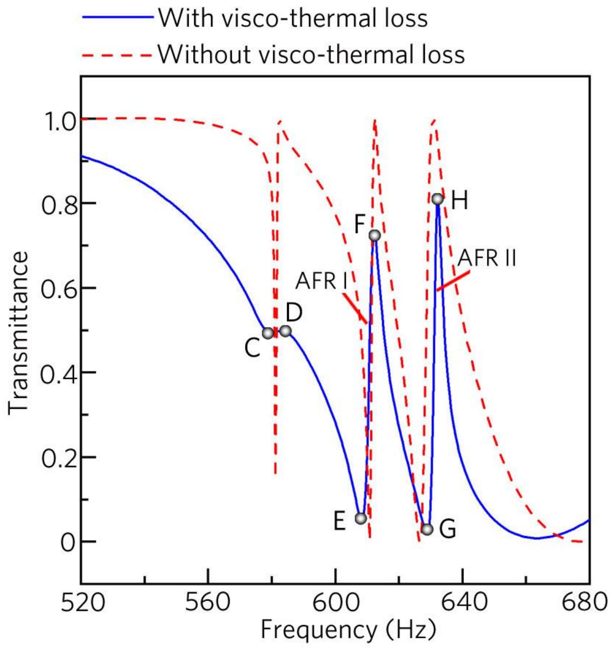

Figure S5. Transmittance spectra of the compound unit array composed of two types of unit cells (r_1=2.7 cm and r_2=2.6 cm) with and without the visco-thermal loss.

Figure S6

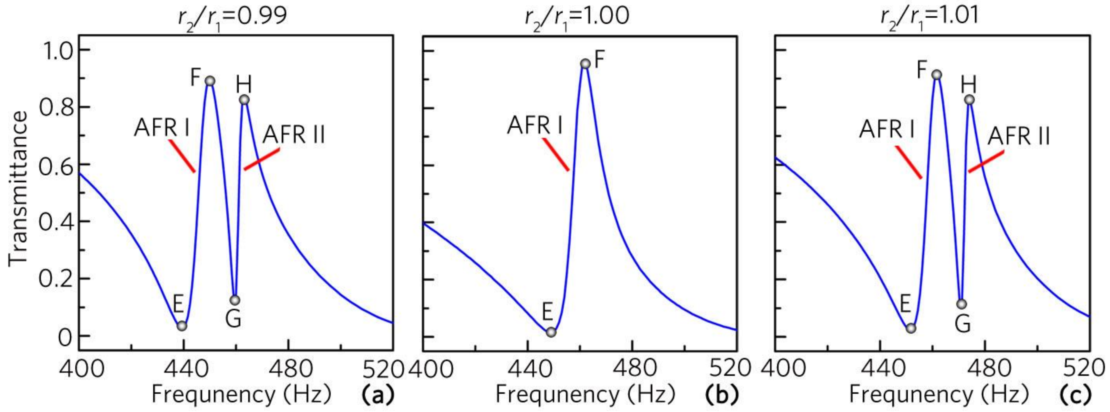

Figure S6. Transmittance spectra of the compound unit arrays composed of two types of unit cells with a) r_2/r_1=0.99, b) r_2/r_1=1.00 and c) r_2/r_1=1.01.
